# Assessment of metabolic patterns and new antitumoral treatment in osteosarcoma xenograft models by [^18^F]FDG and sodium [^18^F]fluoride PET

**DOI:** 10.1186/s12885-018-5122-y

**Published:** 2018-11-29

**Authors:** María Collantes, Naiara Martínez-Vélez, Marta Zalacain, Lucia Marrodán, Margarita Ecay, María José García-Velloso, Marta María Alonso, Ana Patiño-García, Iván Peñuelas

**Affiliations:** 1 Servicio de Medicina Nuclear, Clínica Universidad de Navarra, Avenida Pío XII, 36 31008 Pamplona, Spain; 2 Departamento de Pediatría, Clínica Universidad de Navarra, Avenida Pío XII, 31008 Pamplona, Spain; 3 Small Animal Imaging Research Unit, CIMA, Universidad de Navarra, Avenida Pío XII, 31008 Pamplona, Spain; 4 IdisNA, Instituto de Investigación Sanitaria de Navarra, Pamplona, Spain

**Keywords:** Osteosarcoma, PET, Animal models

## Abstract

**Background:**

Osteosarcoma is the most common malignant bone tumor in children and young adults that produces aberrant osteoid. The aim of this study was to assess the utility of 2-deoxy-2-[18F-] fluoro-D-glucose ([^18^F] FDG) and sodium [^18^F] Fluoride (Na [^18^F] F) PET scans in orthotopic murine models of osteosarcoma to describe the metabolic pattern of the tumors, to detect and diagnose tumors and to evaluate the efficacy of a new treatment based in oncolytic adenoviruses.

**Methods:**

Orthotopic osteosarcoma murine models were created by the injection of 143B and 531MII cell lines. [^18^F]FDG and Na [^18^F] F PET scans were performed 30 days (143B) and 90 days (531MII) post-injection. The antitumor effect of two doses (10^7^ and 10^8^ pfu) of the oncolytic adenovirus VCN-01 was evaluated in 531 MII model by [^18^F] FDG PET studies. [^18^F] FDG uptake was quantified by SUVmax and Total Lesion Glycolysis (TLG) indexes. For Na [^18^F] F, the ratio tumor SUVmax/hip SUVmax was calculated. PET findings were confirmed by histopathological techniques.

**Results:**

The metabolic pattern of tumors was different between both orthotopic models. All tumors showed [^18^F] FDG uptake, with a sensitivity and specificity of 100%. The [^18^F] FDG uptake was significantly higher for the 143B model (*p* < 0.001). Sensitivity for Na [^18^F] F was around 70% in both models, with a specificity of 100%. 531MII tumors showed a heterogeneous Na [^18^F] F uptake, significantly higher than 143B tumors (*p* < 0.01). Importantly, [^18^F] FDG and Na [^18^F] F uptake corresponded to highly cellular or osteoid-rich tumors in the histopathological analysis, respectively. [^18^F] FDG data confirmed that the oncolytic treatment of 531MII tumors produced a significant reduction in growth even with the 10^7^ pfu dose.

**Conclusions:**

PET studies demonstrated that the different osteosarcoma xenograft models developed tumors with diverse metabolic patterns that can be described by multitracer PET studies. Since not all tumors produced abundant osteoid, [^18^F] FDG demonstrated a better sensitivity for tumor detection and was able to quantitatively monitor in vivo response to the oncolytic adenovirus VCN-01.

**Electronic supplementary material:**

The online version of this article (10.1186/s12885-018-5122-y) contains supplementary material, which is available to authorized users.

## Background

Osteosarcoma is the most common primary malignant bone tumor. This aggressive tumor of mesenchymal origin produces aberrant osteoid and is the most frequent skeletal neoplasm in children and adolescents, being the third most common cancer in this age group [1]. The primary tumors mainly occur in the long bones of the extremities near the metaphyseal growth plate and, at initial diagnosis, most patients present micrometastatic disease and an additional 15–20% have visible metastases.

Currently, the therapeutic strategy includes neo- and adjuvant chemotherapy combined with surgical removal of detectable disease [2], but the long-term outcome for patients with detectable metastases is still insufficient. Therefore, multiple new therapeutic approaches have been developed to improve clinical outcome for metastatic patients [3].

In the clinical setting, positron emission tomography (PET) has emerged as a new potent tool in the management of osteosarcoma. The main advantage of PET compared to other structural imaging techniques like MRI or CT is that it can not only detect tumors, but also offer quantitative metabolic information with prognostic value. By large, the glucose analog 2-deoxy-2-[^18^F] fluoro-D-glucose ([^18^F] FDG) is the most commonly used radiotracer to detect tumors, including osteosarcoma [4], based on the increased glucose metabolism of malignant cells. In osteosarcoma, [^18^F] FDG PET/CT helps to detect recurrences in patients with suspicious of relapse after treatment [5], being more sensitive than bone scintigraphy in detecting bone metastases [6]. Moreover a decrease in PET semi-quantitative indexes predicts poor response to treatment even after the first cycle of neoadjuvant chemotherapy in sarcoma and osteosarcoma [7, 8]. Consequently, [^18^F] FDG PET/CT can provide critical information about treatment planning.. In addition to [^18^F] FDG, other PET radiotracers can offer complementary information about the metabolic pattern and phenotype of the tumors. Among these, sodium [^18^F] fluoride (Na [^18^F] F) is of particular interest because it is a positron-emitting bone-seeking agent that mainly reflects remodeling of bone. It can hence be used to image different bone diseases [9] and potentially the osteoblastic mineralization that occurs during osteosarcoma growth. In animal models of osteosarcoma, the combination of [^18^F] FDG and Na [^18^F] FPET radiotracers is able to describe the different metabolic features of tumors, distinguishing between the osteolytic and osteoblastic phenotypes [10–12]. The capacity of PET to describe in vivo and non-invasively the tumor metabolism is therefore of great interest for the study of new treatments in animal models, and in the clinical setting, it could allow in the future to select personalized treatments based on the biologic features of osteosarcomas [13, 14].

The aim of this study was to further explore the ability of PET to detect and describe the metabolic pattern of osteosarcoma tumors, as well as its usefulness to evaluate the efficacy of new treatments. [^18^F] FDG and Na [^18^F] F radiotracers were used in two different orthotopic intratibial mouse models of osteosarcoma, obtained with a commercial (143B) and a primary osteosarcoma-derived (531MII) cell lines. Both radiotracers were employed to describe the metabolic phenotype of tumors and to calculate their sensitivity and specificity for detecting osteosarcomas. In addition, [^18^F]FDG was used to assess the response to different doses of a new experimental treatment based on the oncolytic adenovirus VCN-01 [15].

## Methods

### Cell lines

The human osteosarcoma cell line 143B was obtained from the American Type Culture Collection (ATCC® CRL8303™). Cell line 531MII was developed at the University Clinic of Navarra and corresponds to a metastatic bone implant from an adult patient with a metastatic bone implant. The clinical characteristics and also the molecular hallmarks (*RB1* LOH and *TP53* mutation) of the cell line have been reported previously by our group [16]. Cells were cultured in α-Minimum Essential Medium (a-MEM) supplemented with 10% fetal bovine serum in a humidified atmosphere containing 5% CO_2_ at 37 °C.

### Adenovirus construction and infection

VCN-01 adenovirus presents a modified fiber protein that confers an enhanced infectivity and expresses the human *PH20* gene that encodes for a soluble hyaluronidase that degrades the extracellular matrix of the tumors [15]. Construction and method of infection of oncolytic adenovirus VCN-01 have been described previously [17, 18].

### Animal model and experimental design

**Fig. 1:**
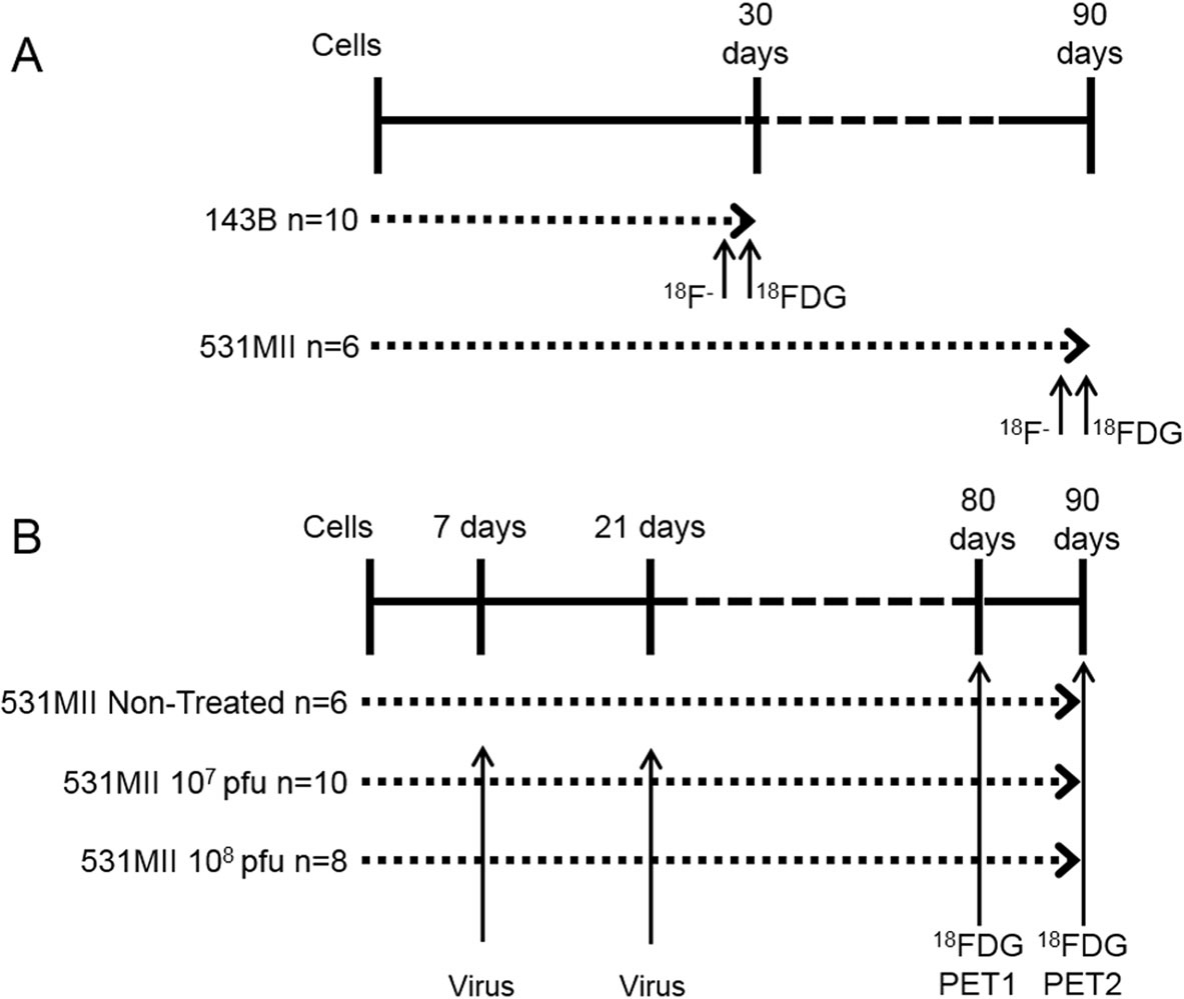
Experimental design**.** Experimental protocol design for (**a**) the detection and description of osteosarcoma in two different experimental orthotopic models using [^18^F]FDG and Na[^18^F]F radiotracers and (**b**) the assessment of a new experimental treatment based on the use of the oncolytic adenovirus VCN-01by [^18^F]FDG. “n” refers the number of tumors analyzed

The experimental design is summarized in Fig. 1. All the procedures involving animals performed in this study were carried out in accordance with the guidelines of the European Community (Council Directive 2010/63/UE) and the Spanish Government (Real Decreto 53/2013) and were approved by the Ethics Committee for Animal Experimentation of the University of Navarra (protocols 065–13 and 160–11).

Female athymic nude mice (NCr nude, Taconic Farms, Inc.), 8–9 weeks of age and between 20 and 22 g were used in this study. Mice were socially housed (4–5 animals per cage) in individually ventilated cages in an air-conditioned room at 22 °C under a 12 h light/dark cycle with access to food and tap water ad libitum during all the experiment, except the day before PET studies (see below).

For the orthotopic intratibial model, mice were injected in the tibial plateau of both hindlimbs with 5 × 10^5^ 143B cells (*n* = 5, 10 hindlimbs) or 531MII (*n* = 3, 6 hindlimbs).

To detect and examine in vivo tumoral metabolic profile (Fig. 1a), [^18^F] FDG and Na [^18^F] F static PET scans were performed in consecutive days, 30 days (143B) or 90 days (531MII) post tumor cell injections, when tumors were macroscopically evident. The day after the last PET scan, animals were euthanized by cervical dislocation and tumors collected for histopathological analysis. As negative control, additional mice without tumors were studied with [^18^F] FDG and Na[^18^F] F (*n* = 2, 4 hindlimbs) that were used to obtain reference values in the quantitative analysis.

To study PET usefulness for treatment assessment (Fig. 1b), other group of mice was injected with 5 × 10^5^ 531MII cells in both hindlimbs. After 7 days, mice were randomized in three groups: non-treated (positive control; *n* = 3, 6 hindlimbs) and treated with 10^7^ (*n* = 5, 10 hindlimbs) or with 10^8^ (*n* = 4, 8 hindlimbs) pfu of oncolytic adenovirus VCN-01. Virus treatment consisted of intratumoral injections 7 and 21 days after cell implantation, whereas non-treated positive control mice received phosphate-buffered saline (PBS) injections. PET studies for treatment monitoring were performed 80 and 90 days post tumor cells inoculation in all animals with [^18^F] FDG. Additionally, two mice without tumors were studied as negative controls. At end point, and after cervical dislocation of the mice, hindlimbs/tumors were excised for histopathological analysis.

### PET acquisition

For PET procedure mice were fasted overnight but allowed to drink water ad libitum. The day of the [^18^F] FDG study, mice were anesthetized with 2% isoflurane in 100% O_2_ gas and [^18^F] FDG (18.3 ± 0,66 MBq in 80–100 μL) was injected via the tail vein. To avoid radiotracer uptake in the hindlimb muscle due to animal movement, [^18^F] FDG uptake was performed under continuous anesthesia for 50 min. For Na [^18^F] F imaging, radiotracer (18.2 ± 1.04 MBq in 80–100 μL) was injected via tail vein in awake mice 90 min before PET study. In all studies, radiotracer uptake was performed with animals over a heating pad heated at 37 °C. PET scans were acquired in a dedicated small animal Philips Mosaic tomograph (Cleveland, OH), with 2 mm resolution, 11.9 cm axial field of view (FOV) and 12.8 cm transaxial FOV. Mice were placed prone on the PET scanner bed to perform a static acquisition (sinogram) of 15 min under anesthesia (isoflurane). Images were reconstructed using the 3D Ramla algorithm (a true 3D reconstruction) with 2 iterations and a relaxation parameter of 0.024 into a 128 × 128 matrix with a 1 mm voxel size applying dead time, decay, random and scattering corrections.

### PET analysis

For PET analysis, all studies were exported and analyzed using the PMOD software (PMOD Technologies Ltd., Adliswil, Switzerland). Images were expressed in standardized uptake value (SUV) units, using the formula SUV = [tissue activity concentration (Bq/cm^3^)/injected dose (Bq)] × body weight (g). An expert observer determined the presence of tumors detected by each radiotracer. After this qualitative detection, a semi-quantitative analysis was performed drawing a volume of interest (VOI) containing the entire hindlimb. For [^18^F] FDG quantitative analysis, maximum voxel intensity within the VOI (SUVmax) was recorded. For Na [^18^F] F evaluation, and in order to normalize tumor uptake respect to physiological uptake in normal bone, a ratio between tumor SUVmax and hip SUVmax was calculated (SUVmax ratio), considering SUVmax of the hip the value of physiological uptake of the radiotracer. For treatment assessment with [^18^F] FDG, in addition to SUVmax values, total lesion glycolysis (TLG) was calculated as the product of mean SUV within the VOI and the tumor volume (cm^3^).

### Ki-67 immunostaining

After sacrifice, hindlimbs/tumors were collected to perform a histopathologic characterization. Paraffin sections (3 μm thick) were cut, dewaxed and hydrated. Sections of all tumors were stained with hematoxylin and eosin for histologic evaluation. Immunohistochemical staining for Ki-67 (rabbit monoclonal, clone SP6, 1:100, NeoMarkers; RM-9106) was performed on those tumors/hindlimbs of treated mice, using the EnVisionTM+ System (Dako, Glostrup, Denmark) according to the manufacturer’s recommendations.

### Statistical analysis

Statistical analysis was performed with STATA software (StataCorp, Texas, USA). For each animal, two tumors were created (one for each hindlimb). Every tumor was considered an independent value in statistical analysis without taking into account intra-animal correlation. Since not all mice developed tumors in both hindlimbs, sensitivity and specificity of [^18^F] FDG and Na [^18^F] F for tumor detection were evaluated comparing qualitative PET results with the presence of primary tumors detected after mice sacrifice only in mice used for orthotropic models. [^18^F] FDG SUVmax and Na [^18^F] F SUVmax ratio values between 531MII and 143B models and negative control animals were compared by the nonparametric Kruskal–Wallis test followed by Bonferroni post hoc test. To evaluate [^18^F] FDG usefulness for treatment assessment, SUVmax and TLG values between negative controls, PBS positive controls and two experimental groups treated with 10^7^ or 10^8^pfu of adenoviruses were analyzed at different time points using the nonparametric Kruskal–Wallis test and Bonferroni post hoc test. Wilcoxon signed-rank test was applied to study differences between both [^18^F] FDG studies in the same group.. All data were expressed as mean ± standard deviation (SD) and *p*-values lower than 0,05 considered as statistically significant.

## Result

### Description of the tumors

**Fig. 2:**
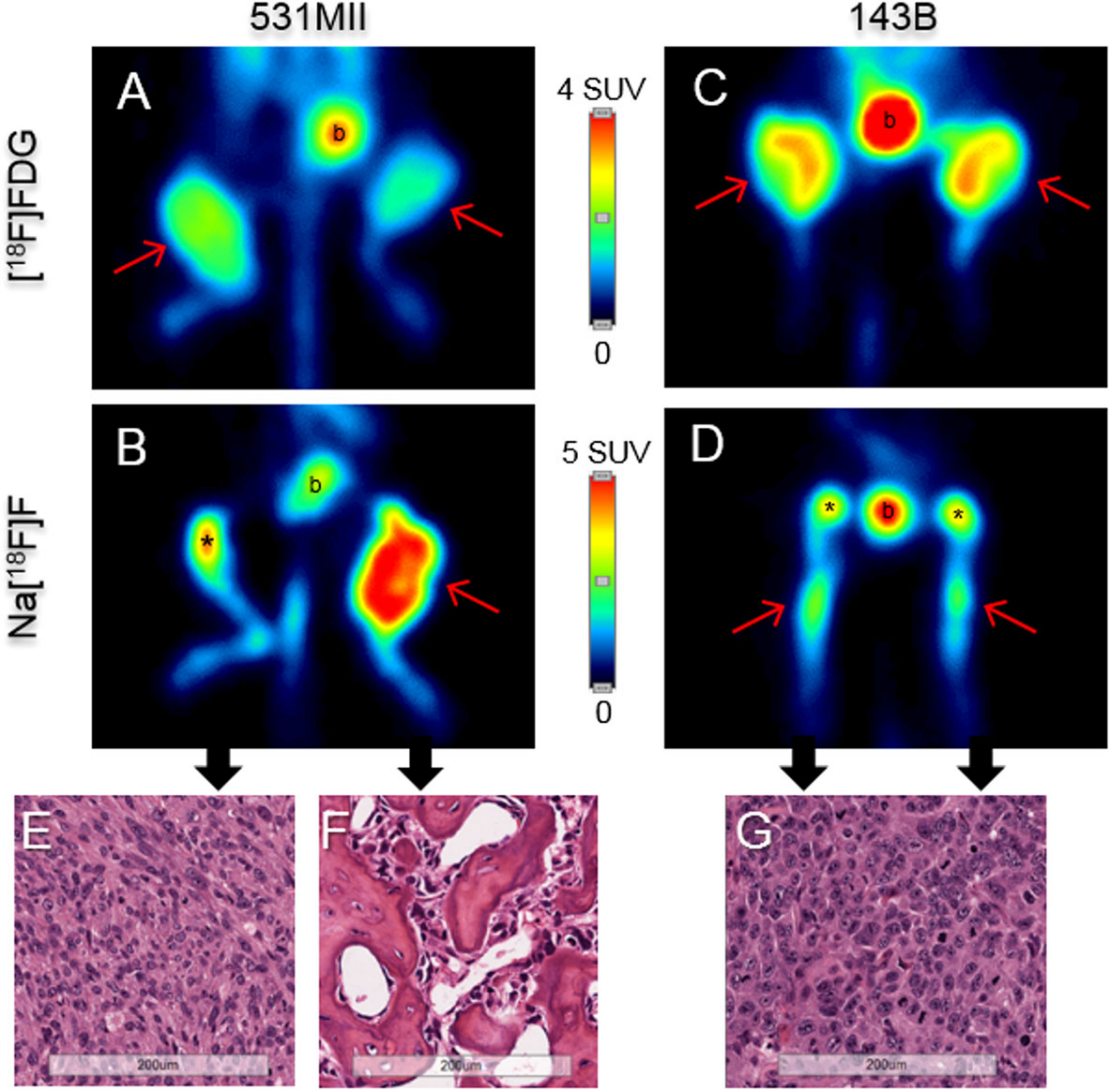
Metabolic description of the tumors using [^18^F]FDG and Na[^18^F]F. Representative PET studies obtained in orthotopic models of osteosarcoma. A and B images correspond to the same animal injected with 531MII cell line, whereas C and D images were obtained from other animal injected with 143B line. The use of [^18^F]FDG (**a, c**) or Na[^18^F]F (**b, c**) allowed detection of different metabolic patterns in the tumors. Images from sections stained with hematoxylin-eosin from each tumor (**e, f, g**) confirmed that the 531MII cell line created heterogeneous tumors with osteoblastic or osteolytic phenotypes, whereas the 143B model generated osteolytic tumors with high cellularity. Red arrows: tumors detected by PET; asterisk: physiological utptake of Na[^18^F]F in knees; b: bladder showing physiological excretion of radiotracer

The use of [^18^F] FDG and Na [^18^F] F allowed to describe the metabolic pattern of tumors, that was different between both orthotopic models (Fig. 2). The cell line 531 MII generated tumors with different phenotypes (*n* = 5), some with high [^18^F] FDG and low Na [^18^F] F uptake and others with the opposite pattern (Fig. 2a, b and Additional file 1: Figure S1). On the other hand, all the tumors from cell line 143B showed a very high [^18^F] FDG signal, but a very slight or no Na [^18^F] F uptake (*n* = 10) (Fig. 2c, d and Additional file 1: Figure S1). Importantly, histopathological analysis confirmed that [^18^F] FDG and Na [^18^F] F uptake corresponded to highly cellular and osteoid-rich tumors, respectively (Fig. 2e, f, g and Additional file 1: Figure S1).

**Fig. 3:**
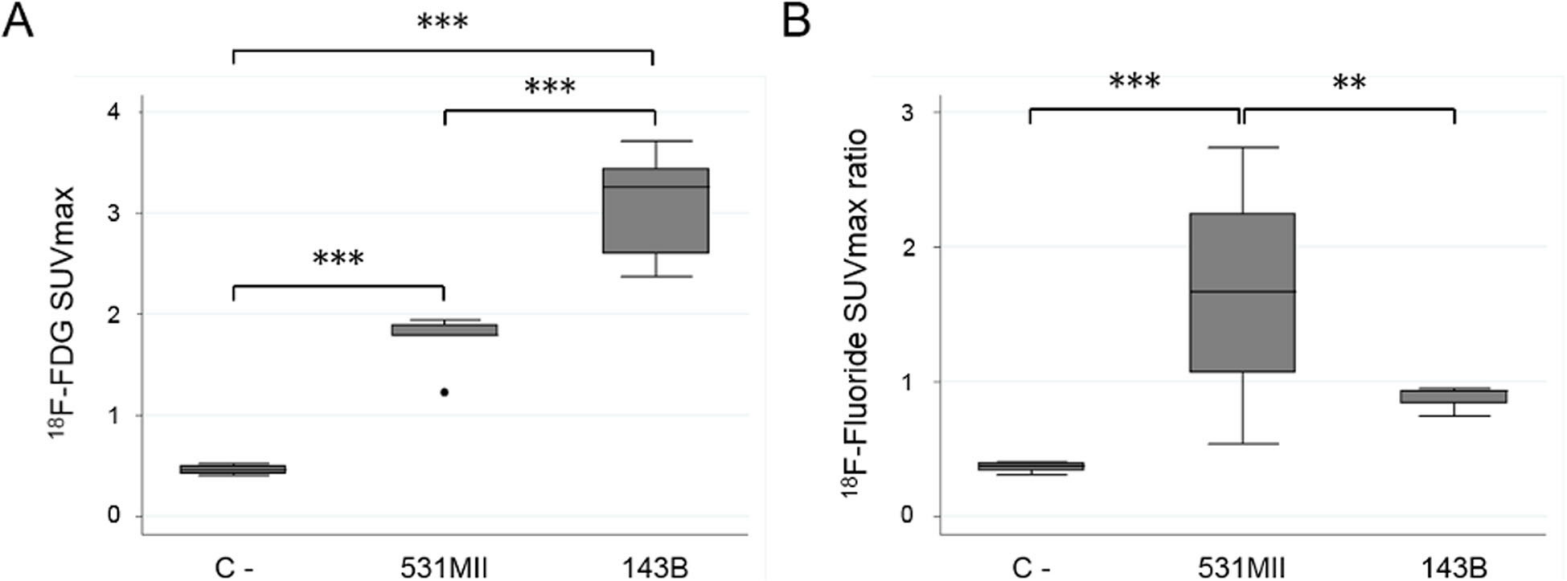
Semi-quantitative analysis of [^18^F]FDG or Na[^18^F]F uptake in osteosarcoma models. Box plots showing quantitative analysis of [^18^F]FDG (**a**) or Na[^18^F]F (**b**) uptake in osteosarcoma models. For each radiotracer, data of semi-quantitative indexes were compared between negative control mice without tumors (C-) and 531MII and 143B tumors. ** *p* < 0.01; ***p < 0.001

Quantitative analysis of the [^18^F] FDG images (Fig. 3a) demonstrated that [^18^F]FDG SUVmax values were statistically significant higher (*p* < 0.001) for both models compared with negative control animals (*n* = 2, 4 hindlimbs) (531MII: 1.73 ± 0.29*;* 143B: 3.08 ± 0.48; C -: 0.46 ± 0.05). In turn, [^18^F] FDG uptake was also significantly higher in 143B than in 531MII tumor models (*p* < 0.001). On the other hand, 531MII tumors showed a heterogeneous Na [^18^F] F uptake, ranging from negative tumors to tumors with very high signal and with SUVmax ratio values higher than in 143B tumors, which showed a very slight signal (531MII: 2.02 ± 0.62 vs. 143B: 0.88 ± 0.09) (Fig. 3b). Differences in Na[^18^F]F values between both models were statistically significant (*p* < 0.01). Only 531MII tumors showed a statistically higher uptake compared with negative control values (0.37 ± 0.04) (p < 0.001), whereas 143B values did not differ from the healthy control (*p* = 0.096).

### Tumor detection

**Fig. 4:**
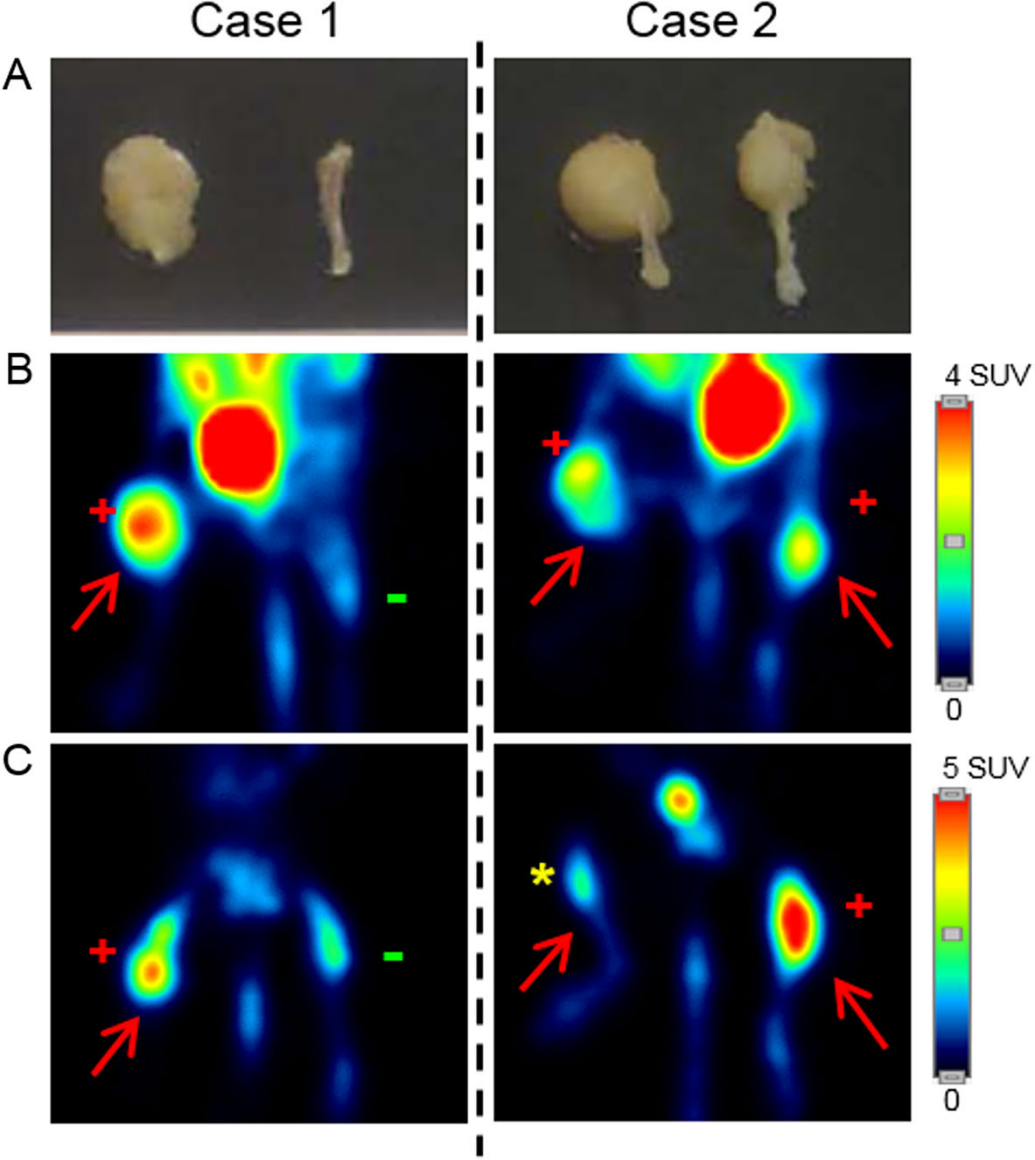
Tumor detection by [^18^F]FDG and Na[^18^F]F. Gross examination of osteosarcoma tumors developed in mice with the 531MII cell line (**a**) and PET images obtained with [^18^F]FDG (**b**) or Na[^18^F]F (**c**). Images on the left and on the right side of the figure correspond to two different animals (case 1 and case 2). PET with [^18^F]FDG clearly distinguished the presence or absence of tumors (red arrows). On the other hand, Na[^18^F]F failed to reveal some tumors. Red positive sign: true positive; green negative sign: true negative; yellow asterisk: false negative

Qualitative analysis of the images showed an excellent correspondence between [^18^F]FDG uptake and gross examination of the tumors (Fig. 4a and b). In this way, in spite of a different degree of uptake, all tumors derived from both cell lines were detected using [^18^F]FDG (531MII, 5/5 tumors and 143B, 10/10 tumors), with a sensitivity and specificity of 100%. However, Na[^18^F]F was not able to detect all osteosarcomas (531MII, 3/5 tumors and 143B, 5/10 tumors), so the sensitivity was around 70% in both models, with a specificity of 100% (Fig. 4a, c).

### Treatment assessment

**Fig. 5:**
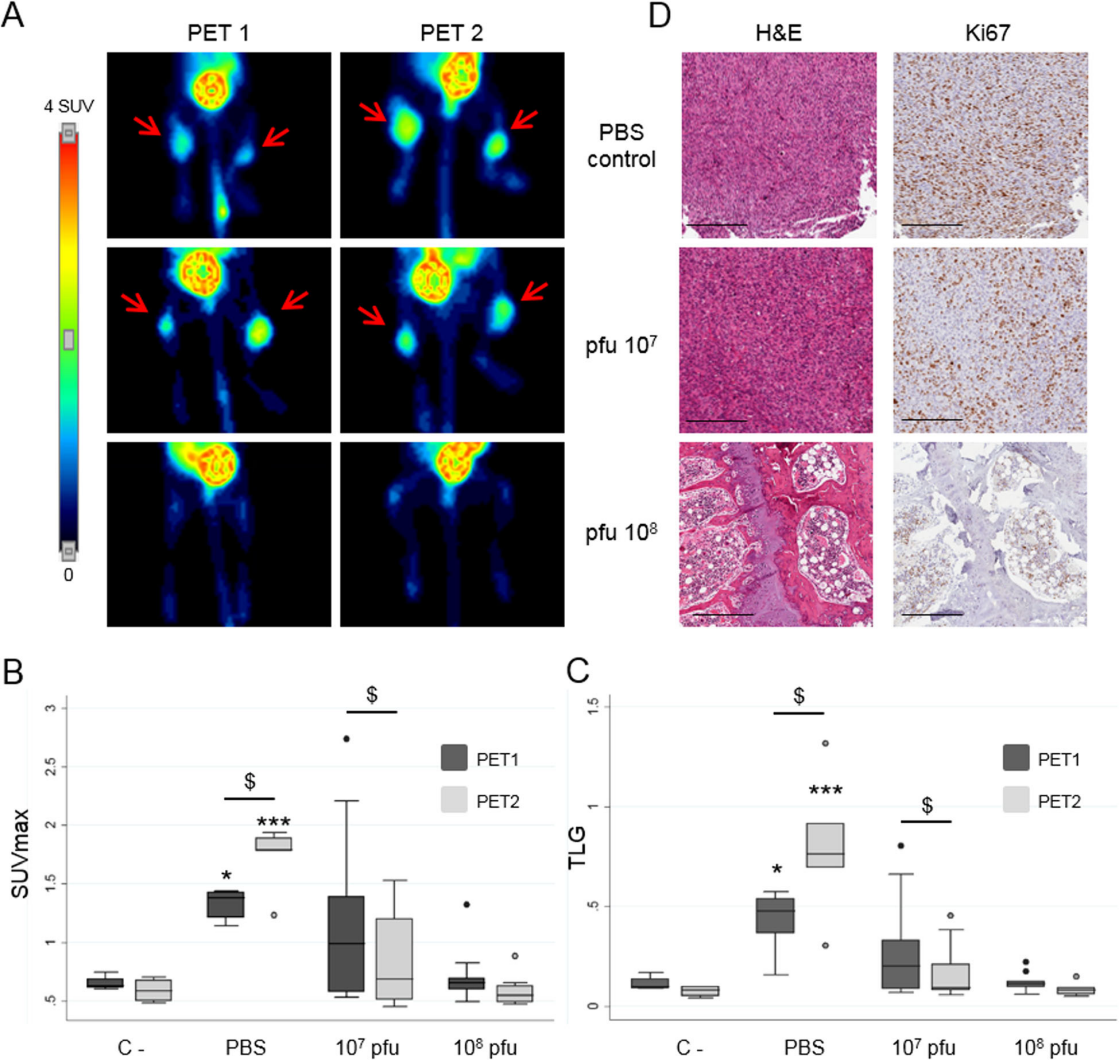
Performance of [^18^F]FDG as PET radiotracer for the evaluation of treatment with the oncolytic adenovirus VCN-01. (**a**) [^18^F]FDG PET images showing tumor evolution in the osteosarcoma model created with 531MII cell line. Images correspond to the non-treated positive control group (PBS control) and the two treatment groups with different adenovirus doses (virus 10^7^ pfu and 10^8^ pfu) studied at two different time points (PET 1 and PET 2). Red arrows point out tumors detected by PET (**b** and **c**). Box plot graphs for quantitative analysis of the [^18^F]FDG images, showing the evolution of SUVmax and TLG data between experimental groups over time and compared with negative controls without tumors (C-). $ symbol represent significant differences between data of the same group in the two time points ($ p < 0,05). * symbols represent statistically significant differences respect C- (* p < 0.05, ***p < 0,001). (**d**) Images of immunohistological analysis corresponding to the right hindlimbs of the same animals showed in (A), and obtained after the sacrifice at the end of the experiment (90 days). Serial sections were stained with hematoxylin and eosin and Ki-67, which detects cellular proliferation. Scale bars, 300 μm

As the sensitivity of [^18^F]FDG was 100% and significantly better than that of Na[^18^F]F, treatment assessment with the oncolytic adenoviruses was monitored in 531MII xenograft model only by [^18^F]FDG studies. PET studies were evaluated at two time points (80 and 90 days after cell implantation) by two semiquantitative indexes, SUVmax and TLG (Fig. 5 and Table 1).

**Table 1:** Treatment assessment. Semi-quantitative analysis of [^18^F]FDG uptake

	SUVmax PET 1	SUVmax PET 2	TLG PET 1	TLG PET 2
C -	0.65 ± 0.62	0.58 ± 0.10	0.11 ± 0.37	0.07 ± 0.27
PBS control	1.32 ± 0.14^*^	1.76 ± 0.29^$,***^	0.42 ± 0.17^*^	0.79 ± 0.37^$,***^
VCN-01 10^7^pfu	1.19 ± 0.75	0.86 ± 0.41^$^	0.27 ± 0.26	0.16 ± 0.14^$^
VCN-01 10^8^ pfu	0.70 ± 0.23	0.58 ± 0.13	0.12 ± 0.48	0.08 ± 0.30

Summary of semi-quantitative analysis of [^18^F]FDG uptake in negative controls (C-), non-treated PBS controls and VCN-01 treated groups (10^7^ and 10^8^ pfu doses) in the different time points (PET 1 and PET 2) after treatment. Data of SUVmax and TLG indexes are shown as mean ± SD. $ symbol represents significant differences between PET 1 and PET 2 in the same group ($ *p* < 0.05). * symbols represent statistically significant differences compared to C- in the same time point (* p < 0.05, ***p < 0.001)

Visual analysis of [^18^F]FDG PET detected tumors only in non-treated mice and the group treated with 10^7^ pfu VCN-01 (Fig. 5a). In the first PET study (day 80), [^18^F]FDG detected tumors in all non-treated mice (*n* = 3, 6 hindlimbs), showing a statistically significant increase in SUVmax and TLG indexes with respect to negative control mice (Table 1 and Fig. 5b and c). No tumors were detected in mice treated 10^8^ pfu, showing a similar [^18^F]FDG uptake to that of negative control animals and pointing out the absence of tumor growth. In the group treated with 10^7^ pfu VCN-01, [^18^F]FDG uptake was heterogeneous, detecting only 3 out of 10 possible tumors. At the end of the experiment (day 90), both TLG and SUVmax indexes in non-treated tumors increased, showing a statistically significant growth as compared with the first PET study (Fig. 5b and c, Table 1). As expected, animals treated with 10^8^ pfu continued to show no sign of [^18^F]FDG uptake. As a group, mice treated with 10^7^ pfu VCN-01 showed decreased [^18^F]FDG uptake and SUVmax and TLG indexes. Gross examination of hindlimbs confirmed that 10^7^ pfu dose exerted a heterogeneous response and that only 3 tumors developed, whereas mice treated with the higher dose of VCN-01 had no evidence of tumors.

Histopathological studies and *Ki-67 immunohistochemistry* at the end of the experiment validated PET findings and confirmed the presence of all tumors detected by [^18^F]FDG PET (Fig. 5d). Hematoxylin and eosin staining showed that the tumors in non-treated mice with a high cellularity also presented a high level of Ki-67 staining, corresponding to an elevated number of proliferating cells. Tumors in the group treated with 10^7^ pfu VCN-01 were similar, albeit with a slight decrease of Ki-67 staining.

## Discussion

This work aimed to confirm the potential of PET metabolic imaging in the study and management of osteosarcomas by studying two orthotopic murine models. In the first part of the study, we confirmed the ability of PET to noninvasively describe the heterogeneous tumor metabolism observed in other similar animal models [10]. For this purpose, multitracer studies have been performed with [^18^F]FDG and Na[^18^F]F radiotracers, which inform respectively about the proliferative state of the tumors and the presence of osteoid or immature bone matrix, a histologic feature of osteosarcomas. The two cell lines used in this study generated two type of tumors with different metabolic characteristics. The commercial cell line 143B produced highly cellular tumors, mimicking osteolytic phenotype, that were detected by [^18^F]FDG showing a very high uptake for this radiotracer, but with very limited or no Na[^18^F]F uptake, thus confirming the results obtained by Campanile et al. [10]. Of great interest are the results obtained with the osteosarcoma cell line 531MII, established from a patient with metastatic disease. This cell line was able to generate tumors with different phenotypes, probably due to clonal selection [19], and hence resembling more closely what occurs in patients. This phenotype diversity, validated in histological studies, was perfectly described in a noninvasive manner by PET imaging. The most osteoblastic tumors showed higher Na[^18^F]F uptake and less [^18^F]FDG uptake, whereas mainly osteolytic tumors presented more avidity for [^18^F]FDG and lower or no uptake of Na[^18^F]F. This ability to describe in vivo tumoral metabolism is a feature of PET imaging that could be employed to personalize the treatments based on the biology of the tumors, a concept behind the new strategies against cancer [20, 21].

Although multitracer studies were able to determine the phenotype of the tumors, it should be noted that only [^18^F]FDG detected all tumors in both models, with a sensitivity and a specificity of 100%. Na[^18^F]F uptake was hampered due to some highly cellular tumors, especially those generated by the 143B cell line, so the sensitivity for this radiotracer was 70%, with a specificity of 100%. This fact may vary at the clinical setting, in which one of the histological hallmarks of osteosarcoma is osteoid production, although in some cases cartilage matrix or fibrous tissue can be predominant [22].

The other main objective of this work was to explore the utility of PET imaging to assess the efficacy of treatment. In the clinical setting, [^18^F]FDG PET is used as an early predictor of response to neoadjuvant chemotherapy in osteosarcoma and other type of tumors [8, 23], and this ability could be used by researchers to monitor the response of new experimental treatments in animal models in a noninvasive manner. In this case, the excellent sensitivity of [^18^F]FDG in our animal models prompted us to use this radiotracer to assess the effect of the oncolytic adenovirus VCN-01 in the 531MII orthotopic model. Oncolytic adenoviruses are genetically modified to infect and destroy the tumor cells in a selective manner without affecting other tissues [24]. In particular, VCN-01 adenovirus replicate in tumors with a defective RB1 pathway and enhance its infectivity through a modified fiber and the expression of a soluble hyaluronidase that degrades extracellular matrix. This new and promising therapy has previously been studied by our group in orthotopic and lung metastatic mice models of osteosarcoma, demonstrating that it exerts a significant and potent antitumor effect in both models [15].

PET [^18^F]FDG studies allowed the monitoring of the response to this therapy in the orthotopic model. Visually, the first image study performed at day 80 demonstrated that the virus produced an antitumor effect in a dose-dependent manner. Interestingly, in this point-time, PET showed that no tumors had been developed in animals treated with VCN-01 at 10^8^ pfu, whereas the low dose of 10^7^ pfu was able to avoid tumor growth only in some cases. The second PET study, performed at the end of the experiment (day 90), confirmed the growth of the tumors in the non-treated group and a decrease in the signal in the tumors treated with 10^7^ pfu. As previously published by Martinez-Vélez et al. [15], molecular techniques demonstrated that the antitumor effect observed in treated groups was caused by the action of VCN-01. Expression of the adenovirus fiber and PH20 mRNA were detected in mice treated with VCN-01, and this expression was higher in tibiae treated with 10^8^ pfu than those treated with 10^7^ pfu. Moreover, viral presence was demonstrated at the end of the experiment, so the decrease in [^18^F]FDG uptake between the two point-times in the 10^7^ pfu treated group could be showing that the action of the adenovirus continues along time, even when treatment was administered long time before (months). The increase over time in VCN-01 viral genome levels in tumor samples after a single administration of adenovirus has previously been demonstrated in other animal models [25].

[^18^F]FDG images of the tumors were quantitatively analyzed by means of two semi-quantitative indexes, SUVmax and TLG. SUVmax reflects the highest metabolic activity of the tumor, whereas TLG represents the overall metabolism of the malignant lesion, combining information about the volume and the metabolic activity of the tumor. Some authors suggest that TLG reflects more accurately the real tumor burden [26, 27] and could be considered as a better prognostic measure than SUVmax. In this model, both SUVmax and TLG indexes increased overtime in tumors of the non-treated group, (reflecting the growth of tumors), whereas in the group treated with 10^7^ pfu VCN-01 such indexes decreased because the treatment continued its effect along time. Due to the small number of tumors obtained after the treatment with VCN-01, further studies are needed to explore the utility of these indexes and how they reflect the mechanism of action of the treatments. Finally, although in this study Na[^18^F]F has been discarded for treatment evaluation, it could be of great interest to include this radiotracer in future studies to ascertain whether hyaluronidase produced by VCN-01 is able to degrade extracellular osteoid and change the metabolic profile of tumors from osteoblastic to a more osteolytic phenotype.

## Conclusions

In conclusion, this study demonstrates that PET studies with [^18^F]FDG and Na[^18^F]F can describe in vivo osteosarcoma phenotypes since the metabolic patterns correlate with the histological appearance of the tumors. We have also proven in vivo that different orthotopic murine models of osteosarcoma lead to tumors with diverse metabolic patterns, and that the cell line 531MII is able to generate tumors with different phenotypes. Since in those osteosarcoma models not all tumors produced abundant osteoid, [^18^F]FDG demonstrated a better sensitivity for tumor detection. This tracer could monitor and prove the treatment efficacy of the oncolytic adenovirus VCN-01, that avoided tumor establishment and growth in a dose dependent manner. Further studies are needed to explore the PET usefulness in the management of osteosarcomas in the clinical setting.

## Additional file

Additional file 1: **Figure S1.** Metabolic description of the tumors using [^18^F]FDG and Na[^18^F]F. Three cases of tumors obtained in orthotopic models of osteosarcoma. Figure shows PET studies from the same animal performed with [^18^F]FDG and Na[^18^F]F, as well as sections of the histopathological analysis stained with hematoxylin-eosin. PET images show the animal in supine position, so the right tibia is to the left of the image and vice versa. Red arrows: tumors detected by PET; asterisk: physiological utptake of Na[^18^F]F in knees; b: bladder showing physiological excretion of radiotracer. (TIF 4289 kb)

## Abbrevations

^18^F-FDG: 2-deoxy-2-[18F]fluoro-D-glucose.
PBS: Phosphate buffered saline.
PET: Positron emission tomography.
pfu: Plaque-forming unit.
SUV: Standardized uptake value.
SUVmax: Maximum standardized uptake value.
TLG: Total lesion glycolysis.
