# Supplementary figures and images for: Assessment of metabolic patterns and new antitumoral treatment in osteosarcoma xenograft models by [18F]FDG and sodium [18F]fluoride PET
Source: BMC Cancer. 2018 Nov 29;18:1193. doi: 10.1186/s12885-018-5122-y (PMC6267920; doi:10.1186/s12885-018-5122-y)

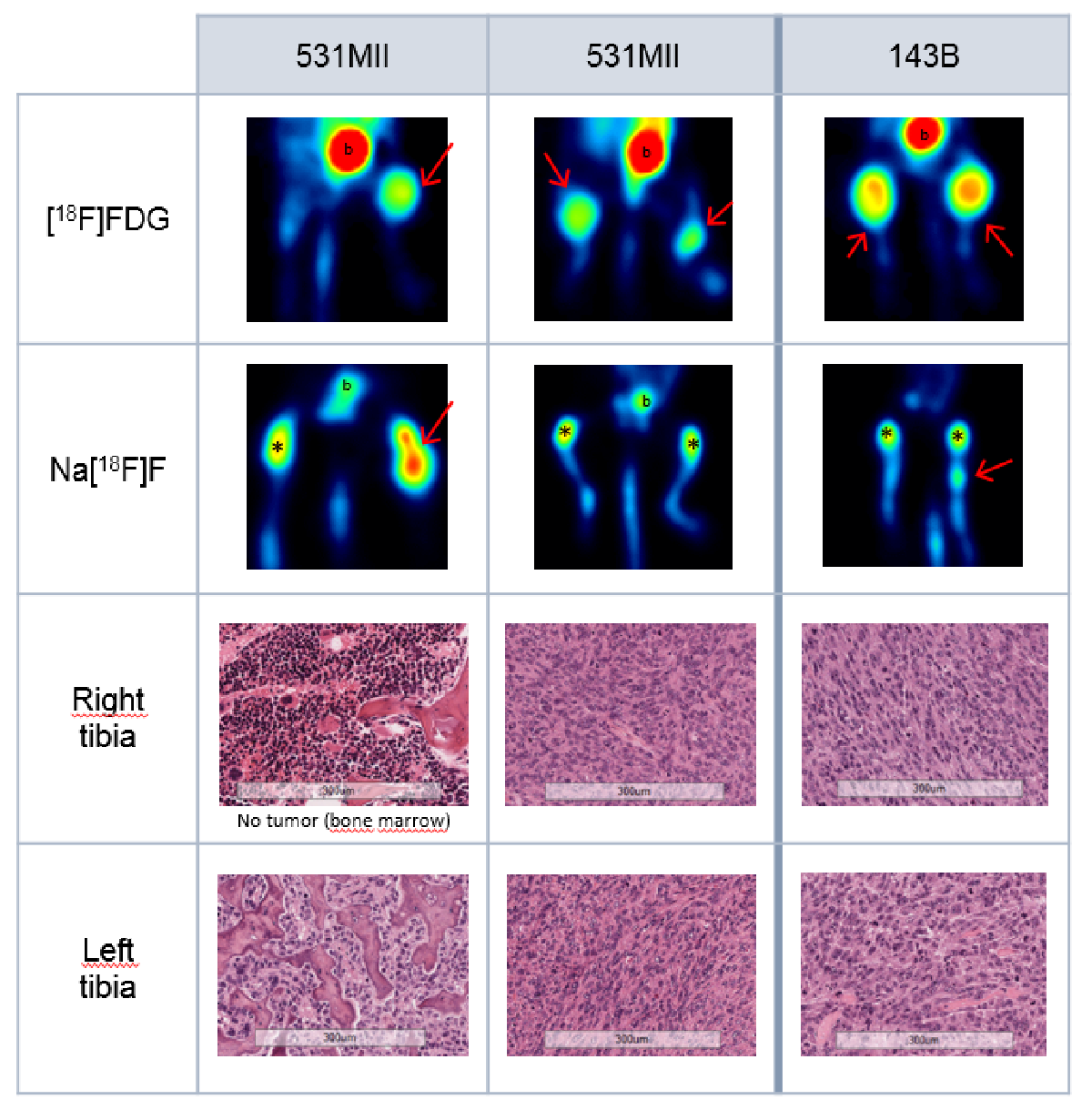

Supplement: Supplementary file 1 — Figure S1. Metabolic description of the tumors using [18F]FDG and Na[18F]F. Three cases of tumors obtained in orthotopic models of osteosarcoma. Figure shows PET studies from the same animal performed with [18F]FDG and Na[18F]F, as well as sections of the histopathological analysis stained with hematoxylin-eosin. PET images show the animal in supine position, so the right tibia is to the left of the image and vice versa. Red arrows: tumors detected by PET; asterisk: physiological utptake of Na[18F]F in knees; b: bladder showing physiological excretion of radiotracer. (TIF 4289 kb) [file 12885_2018_5122_MOESM1_ESM.tif]
